# Correlation between Forced Vital Capacity and the Severity of Frailty-Induced Dysphagia

**DOI:** 10.3390/jcm11071962

**Published:** 2022-04-01

**Authors:** Byung Joo Lee, Sang Cheol Lee, Ho Yong Choi, Min Cheol Chang, Donghwi Park

**Affiliations:** 1 Department of Rehabilitation Medicine, Daegu Fatima Hospital, Daegu 41199, Korea; bjl84@naver.com; 2 Department of Physical Medicine and Rehabilitation, Ulsan University Hospital, University of Ulsan College of Medicine, Ulsan 44033, Korea; 0734587@uuh.ulsan.kr; 3 Department of Neurosurgery, Kyung Hee University Hospital at Gangdong, Kyung Hee University College of Medicine, Seoul 05278, Korea; heoryong83@hanmail.net; 4 Department of Rehabilitation Medicine, Yeungnam University Hospital, Daegu 42415, Korea

**Keywords:** deglutition, dysphagia, pulmonary function test, modified version of VDS, VDS, mVDS

## Abstract

Introduction: Frailty syndrome is a complex condition characterized by the gradual deterioration of an individual’s physical, mental, and social functions. Dysphagia is a dysfunction triggered by frailty. However, in patients with frailty syndrome, dysphagia is often undermined, and a proper evaluation is not performed. Therefore, we tried to identify the factors that can provide proper information regarding dysphagia in the frail population. Methods: Patients with dysphagia were divided into those with frailty-induced dysphagia and those with brain-lesion-induced dysphagia. Factors related to the participants’ pulmonary function test (PFT) results were evaluated. The severity of dysphagia was evaluated by determining modified videofluoroscopic dysphagia scale (mVDS) and penetration–aspiration scale (PAS) scores based on videofluoroscopic swallowing studies. Statistical analysis was performed to determine the correlation between PFT results and the parameters indicating dysphagia severity. Results: Multivariate logistic regression analysis revealed that forced vital capacity (FVC) was significantly correlated with mVDS scores in frailty-induced dysphagia (*p* < 0.05). However, no such significance was detected in brain-lesion-induced dysphagia (*p* ≥ 0.05). Conclusion: FVC was correlated with the severity of dysphagia (mVDS scores) in patients with frailty-induced dysphagia. Thus, serial FVC-based follow-up can be helpful for understanding patients’ dysphagia status. However, studies with a general population of patients with frailty-induced dysphagia are needed for definite generalization.

## 1. Introduction

Frailty syndrome is a complex condition characterized by the gradual deterioration of an individual’s physical, mental, and social functions [1,2]. It is triggered by an aging-related decline in multiple physiological systems [3]. Thus, its prevalence increases with age, and physiological dysfunction due to frailty syndrome often results in disability, hospital admission, and death.

Frailty syndrome is also associated with deterioration of swallowing function. The exact prevalence of dysphagia in frailty syndrome has not been investigated previously. Nevertheless, many studies have reported that frailty is one of the main causes of dysphagia [4,5,6]. However, many clinicians lack knowledge of the relationship between frailty and dysphagia. Therefore, even if dysphagia is present in frail patients, it is often neglected by clinicians, which can increase the prevalence of aspiration pneumonia in frail populations. A videofluoroscopic swallowing study (VFSS) is the gold standard for diagnosing dysphagia [7,8]. However, the lack of facilities for VFSS makes it difficult to perform routine VFSS in patients with frailty-induced dysphagia. In addition, many clinicians who are not dysphagia-related specialists are unable to perform VFSS and interpret the results of these studies. Furthermore, clinicians can be exposed to large doses of radiation, and contrast-induced aspiration is an adverse effect of VFSS [9]. Therefore, clinical parameters that reflect the degree of dysphagia can be helpful to determine whether clinicians should conduct VFSS or refer patients to an expert.

Precise coordination between breathing and swallowing is an important mechanism for preventing pulmonary aspiration [10,11]. Moreover, factors that alter breathing patterns and ventilation, such as chronic respiratory diseases, can influence the precise coordination of breathing and swallowing [12]. Therefore, we hypothesized that several clinical parameters related to breathing are correlated with swallowing function. Moreover, evaluation of clinical parameters related to breathing may be helpful for the early detection and prognostication of dysphagia. Therefore, in this study, we aimed to identify the correlations between respiratory-related parameters (i.e., the results of pulmonary function tests [PFTs]) and the severity of dysphagia in the frail population.

## 2. Materials and Methods

### 2.1. Participants

Data on patients with frailty-induced and brain-lesion-induced dysphagia who underwent VFSS at Ulsan University Hospital between March 2020 and February 2022 were retrospectively collected.

The inclusion criteria for the frailty-induced dysphagia group were as follows: (1) age at VFSS > 50 years; (2) a history of aspiration symptoms, such as coughing or choking; (3) the presence of symptoms clinically indicative of dysphagia, such as reduced gag reflex or delayed swallowing reflex; and (4) the presence of oropharyngeal dysphagia due to frailty or deconditioning without a specific diagnosis that could cause dysphagia, such as stroke, traumatic brain injury, or other laryngeal pathologies [5]. The exclusion criteria for the frailty-induced dysphagia were as follows: (1) esophageal dysphagia; (2) dysphagia due to known neurologic conditions including stroke, traumatic brain injury, anoxic brain injury, brain tumor, amyotrophic lateral sclerosis, Parkinson’s disease, or Alzheimer’s disease; (3) dysphagia from laryngeal pathology, including laryngeal cancer, stenosis, paralysis, and postoperative head and neck surgery [5].

The inclusion criteria for patients with brain-lesion-induced dysphagia were as follows: (1) age at VFSS > 50 years; (2) a history of aspiration symptoms, such as coughing or choking; (3) the presence of symptoms clinically indicative of dysphagia, such as reduced gag reflex or delayed swallowing reflex, occurs newly after brain lesions; and (4) the presence of brain lesions on brain magnetic resonance imaging (MRI) or computed tomography (CT) that could cause dysphagia, such as stroke, traumatic brain injury, or brain cancer. 

Data from the frailty-induced dysphagia group and patients with brain-lesion-induced dysphagia were analyzed. Data on patient age, sex, body mass index (BMI), history of procedures, such as tracheostomy tube, history of using alternative feeding methods, such as a nasogastric tube, forced vital capacity (FVC), forced expiratory volume in one second (FEV1), forced expiratory volume in one second/forced vital capacity (FEV1/FVC), a modified version of videofluoroscopic dysphagia scale (mVDS) score, and penetration–aspiration scale (PAS) score were collected [13].

### 2.2. Protocol for VFSS

VFSS was performed using a fluoroscopy device and recorded in a video file. During the VFSS, the patient swallowed the following substances in order: water, nectar (51–350 cP), rice porridge (351–1750 cP), and boiled rice (>1750 cP) [14,15,16]. The patient swallowed the substances in a sitting position, and all substances were mixed with barium. Dynamic fluoroscopy images were recorded at a rate of 30 frames per second and were obtained from the lateral views. The results were analyzed in terms of PAS and mVDS scores; aspiration was considered present when the PAS score was >5. The mVDS is a scale of 100 points, which is scored by a physiatrist based on several parameters in the VFSS video [14,15,16]. The parameters of the mVDS are listed in Table 1. Previous studies have demonstrated the inter-and intra-reliability of the Mvds [14,15,16].

The images obtained in VFSS were reviewed by two physicians with more than 10 years of experience in interpreting these results. Patient information, including sex, age, and cause of dysphagia, was not provided to the interpreters. The interpreters analyzed the video files to interpret the VFSS results and, accordingly, measured PAS and mVDS scores as parameters of dysphagia severity.

### 2.3. Pulmonary Function Test

Spirometry was conducted according to American Thoracic Society guidelines (Vmax 22, SensorMedics; PFDX, MedGraphics) [17,18]. The following values were evaluated: FVC, forced FEV1, the ratio of FEV1 to FVC (FEV1/FVC). All spirometric values were expressed as percentages of predicted values (percentage of predicted) [17,18].

### 2.4. Sample Size Calculation

The correlation coefficient between FVC value (%, predicted) and mVDS from the 60 of frailty-induced dysphagia group used for sample size calculation, with a standard deviation (SD) of 0.2, significance level of 0.05, and power of 80%.

### 2.5. Statistical Analysis

For comparisons of the patient characteristics between the two dysphagia groups (age, sex, BMI, the prevalence of Levin tube and tracheostomy, pulmonary function test, mVDS, and PAS), *p*-values were also calculated by an independent Pearson Chi-square test or an independent *t*-test as appropriate.

The correlations between the mVDS score and pulmonary function test (FEV1, FVC, and FEV1/FVC) results were verified in the patients with frailty-induced dysphagia and those with brain-lesion-induced dysphagia. For this, multivariate regression analysis using the enter method was used to evaluate the correlation between mVDS score and FVC, mVDS score and FEV1/FVC, PAS score and FVC, and PAS score and FEV1/FVC in each group. FEV1 was excluded from both multivariate regression analyses due to multicollinearity with FVC (VIF = 11.971). Statistical analyses were performed using MedCalc and SPSS software (version 22.0; IBM, Armonk, NY, USA).

## 3. Results

### 3.1. Patient Characteristics

Based on the sample size calculation, the required sample size for this study was at least 51 participants in each group. Therefore, a total of 114 patients with dysphagia (91 men, 23 women) participated in this study. Among them, 60 patients were classified into the frailty-induced dysphagia group, and the remaining 54 patients were classified into the brain-lesion-induced dysphagia group. Between the two groups, there were statistically significant differences were observed in the results of BMI and the prevalence of Levin-tube (*p* < 0.05) (Table 2). However, other characteristics, such as age, sex, and prevalence of tracheostomy, there was no statistically significant difference observed between the two groups (*p* ≥ 0.05) (Table 2). In the PFTs, there were no statistically significant differences were observed in the results of PFTs between the two groups (*p* ≥ 0.05) (Table 2).

In the VFSS test, although the PAS scores differed significantly between the two groups, the mVDS scores did not differ significantly between the two groups (Table 2). Patient statistical data are presented in Table 2.

The classification of patients with brain-lesion-induced dysphagia is presented in Table 3.

### 3.2. Patients with Brain-Lesion-Induced Dysphagia 

Multivariate regression analysis showed no significant correlation of mVDS scores with FVC and FEV1/FVC in the brain-lesion-induced dysphagia group (*p* > 0.05) (Table 4). In addition, there was no significant correlation between PAS scores and FVC and FEV1/FVC in the brain-lesion-induced dysphagia group (*p* ≥ 0.05) (Table 4).

### 3.3. Patients with Frailty-Induced Dysphagia

Multivariate regression analysis showed that the mVDS score was only significantly correlated with FVC in the frailty-induced dysphagia group (*p* < 0.05) (Table 5). The value of FVC and the severity of dysphagia (mVDS) were inversely proportional. However, the mVDS score was not significantly correlated with FEV1/FVC (Table 5). Multivariate regression analysis showed that the PAS score was not significantly correlated with FVC and FEV1/FVC in the frailty-induced dysphagia group (*p* ≥ 0.05) (Table 5).

## 4. Discussion

In the results of this study, FVC was significantly correlated with mVDS scores, which indicate the severity of dysphagia in the frailty-induced dysphagia group. However, this significance was not detected in the patients with brain-lesion-induced dysphagia. 

In this study, we compared the pulmonary function test with the severity of dysphagia because we hypothesized that several clinical parameters related to breathing are correlated with swallowing function. Moreover, evaluation of clinical parameters related to breathing may be helpful for the early detection and prognostication of dysphagia in the frailty-induced dysphagia group. We also included patients with brain-lesion-induced dysphagia as controls, as central nervous system (CNS) damage such as brain lesions is one of the most common causes of dysphagia [19]. Since the pulmonary function test using spirometry is widely used in research as well as clinical trials, it is a useful indicator regardless of location as a portable device for measurement. In this study, spirometric parameters such as FEV1, FVC, and FEV1/FVC were used [20].

Previous studies have reported that FVC is correlated with inspiratory muscle power [21,22]. Similar to the limb muscles, the inspiratory and swallowing muscles can also be affected by frailty, and because of the proximity of the anatomical locations of these inspiratory and swallowing muscles, dysphagia severity and FVC are thought to be correlated in patients with frailty-induced dysphagia in this study [6].

These results can be explained by several reasons, which are presented below. First, precise coordination between breathing and swallowing is important for preventing pulmonary aspiration [23]. Although the exact neural processes by which these behaviors (breathing and swallowing) are coordinated are not well understood, it is hypothesized that swallowing and breathing are coordinated to occur at specific times relative to one another to minimize the risk of aspiration by a common control system present in the brain stem [24]. Therefore, PFT parameters, especially FVC, which can represent breathing and ventilation to some extent, may have been associated with the severity of dysphagia. Second, muscle weakness due to frailty is not limited to one muscle; all muscles, including the limb, respiratory, and swallowing muscles, can be affected. Therefore, FVC, which is known to correlate with respiratory muscle power, may have a significant correlation with the severity of dysphagia, which is proportional to the weakening of swallowing muscle power. However, in the brain-lesion-induced dysphagia group, other factors, such as neurologic deterioration, could have additional effects on the outcomes and could therefore have hindered the relationship between FVC and the severity of dysphagia. 

To our knowledge, no previous study has investigated the correlation between mVDS scores and FVC in patients with frailty-induced dysphagia. In patients with frailty-induced dysphagia, dysphagia is often neglected, and VFSS cannot be performed in many cases, although VFSS remains the gold standard for the diagnosis of dysphagia. The results of this study suggest that FVC can reflect the severity of dysphagia, especially in patients with frailty. Clinicians should suspect the presence of dysphagia in frail patients with a low FVC, and dysphagia evaluations such as VFSS should be recommended in such patients. Moreover, since FVC measurements can be easily obtained with a portable device, serial measurement of FVC in patients with frailty could be helpful in evaluating the progression of dysphagia severity without the risk of contrast-induced aspiration pneumonia and radiation exposure. However, further studies with a larger number of patients with frailty-induced dysphagia are needed for a more definite generalization. 

Our study had several limitations. First, it was a retrospective study. Therefore, data for factors that could have yielded more detailed results, such as clinical characteristics of dysphagia from a valid questionnaire, maximum inspiratory pressure (MIP), or maximum expiratory pressure (MEP), were not collectable. Since MIP and MEP are more strongly correlated with respiratory muscle power than FVC, future studies using MIP and MEP may show interesting results for the association between dysphagia severity and these indicators. Second, the number of participants in this study was relatively small. Further studies with larger sample sizes are needed for more accurate generalization. Third, we could not clarify whether chronic lung disease was included in this study. This is because, due to the characteristics of the retrospective study, not all patients were diagnosed whether all patients had chronic lung disease by respiratory specialists. Therefore, some patients with chronic lung disease may have been enrolled in both the frailty-induced and brain-lesion-induced dysphagia groups. However, there was no statistically significant difference between the two groups in FEV1/FVC and FVC, which are important diagnostic criteria for chronic lung disease. Therefore, the inclusion of some chronic lung disease patients in both groups is not expected to significantly affect the results of this study. However, further research is needed in the future. Lastly, this study was conducted at a single tertiary hospital, and future multicenter studies can address the potential bias in the findings reported in this study.

## 5. Conclusions

FVC was correlated with mVDS scores in patients with frailty-induced dysphagia. Routine pulmonary function testing, especially FVC evaluations, can be helpful in understanding the status of dysphagia. However, studies with a general population of patients with frailty-induced dysphagia are needed for evaluating the generalizability of the findings.

## Figures and Tables

**Table 1:** Modified version of the videofluoroscopic dysphagia scale.

Parameters		Score
Lip closure	Intact/not intact	0/6
Mastication	Possible/not possible	0/11.5
Oral transit time	≤1.5 s/>1.5 s	0/4
Triggering pharyngeal swallow (swallowing reflex)	Intact/delayed	0/7
Epiglottis inversion	Yes/no	0/13
Valleculae residue	0%/<10%/≥10%, <50%/≥50%	0/3/6/9
Pyriformis residue	0%/<10%/≥10%, <50%/≥50%	0/6.5/13/19.5
Pharyngeal wall coating	No/yes	0/13
Aspiration	Intact/penetration/aspiration	0/8.5/17
Total score		100

**Table 2:** Clinical characteristics of the patients with dysphagia who were included in this study.

Variable	Total	Brain Lesions	Frailty	*p*-Value
Total, *n* (%)	114 (100%)	54 (47%)	60 (53%)	
Age, years	71.20 ± 10.67	73.17 ± 8.69	69.38 ± 11.96	0.090
Gender, *n* (%)				
Female	23/114 (20%)	7/54 (13%)	16/60 (26.7%)	0.069
Male	91/114 (80%)	47/54 (87%)	44/60 (73.3%)	
BMI (kg/m^2^)	19.58 ± 3.99	18.80 ± 3.58	20.28 ± 4.23	0.049 *
T-tube	24/114 (21%)	12/54 (22.2%)	12/60 (20.0%)	0.771
L-tube	52/114 (46%)	32/54 (59.3%)	20/60 (33.3%)	0.006 *
Pulmonary function test				
FVC (% predicted)	50.75 ± 24.83	51.63 ± 22.91	50.20 ± 24.50	0.913
FEV1 (% predicted)	54.33 ± 22.41	51.45 ± 23.44	56.73 ± 21.47	0.310
FEV1/FVC ratio	79.75 ± 14.20	81.33 ± 14.65	77.28 ± 16.14	0.259
VFSS				
mVDS score	42.04 ± 22.14	43.71 ± 21.63	40.54 ± 22.67	0.396
PAS	4.52 ± 3.19	5.31 ± 3.25	3.80 ± 2.97	0.032 *

* Significant difference was noted between the two groups (*p* < 0.05). Values are presented as mean ± standard deviation. T-tube, tracheostomy tube; L-tube, Levin tube; BMI, body mass index; FVC, forced vital capacity; FEV1, forced expiratory volume in one second; FEV1/FVC, forced expiratory volume in one second/forced vital capacity; VFSS, videofluoroscopic swallowing study; mVDS, modified videofluoroscopic dysphagia scale; PAS, penetration–aspiration scale.

**Table 3:** Classification of patients with brain-lesion-induced dysphagia.

	No. of Patients
Total, *n* (%)	54 (100%)
Cerebral infarction	35 (64.81%)
Cerebral hemorrhage	1 (1.85%)
Traumatic brain injury	6 (11.11%)
Meningitis	3 (5.56%)
Brain tumor	9 (16.67%)

**Table 4:** Multivariate regression analysis (with the enter method) of the association between the penetration–aspiration scale (PAS) scores and PFT (FVC and FEV1/FVC), between the modified version of the videofluoroscopic dysphagia scale (mVDS) scores and PFT (FVC and FEV1/FVC) in the patients with brain-lesion-induced dysphagia.

		Unstandardized Coefficients		Standardized Coefficients			95% CI		Collinearity Statistics	
	Parameter	B	Standard Error	Beta	t	Parameter	B	Standard Error	Tolerance	VIF
FEV1/FVC	PAS	−0.008	0.035	−0.035	−0.214	PAS	−0.008	0.035	0.991	1.009
FVC	PAS	−0.016	0.022	−0.119	−0.718	PAS	−0.016	0.022	0.991	1.009
FEV1/FVC	mVDS	0.231	0.235	0.162	0.985	mVDS	0.231	0.235	0.991	1.009
FVC	mVDS	0.049	0.150	0.053	0.323	mVDS	0.049	0.150	0.991	1.009

CI: Confidence interval. PFT, pulmonary function test; FVC, forced vital capacity; FEV1/FVC, forced expiratory volume in one second/forced vital capacity.

**Table 5:** Multivariate regression analysis (with the enter method) of the association between the penetration–aspiration scale (PAS) scores and PFT (FVC and FEV1/FVC) and between the modified version of the videofluoroscopic dysphagia scale (mVDS) scores and PFT (FVC and FEV1/FVC) in the patients with frailty-induced dysphagia.

		Unstandardized Coefficients		Standardized Coefficients			95% CI		Collinearity Statistics	
	Parameter	B	Standard Error	Beta	t	*p*-Value	Lower Bound	Upper Bound	Tolerance	VIF
FEV1/FVC	PAS	0.043	0.033	0.227	1.288	0.206	−5.014	0.110	1.000	1.000
FVC	PAS	−0.018	0.022	−0.142	−0.807	0.425	−0.062	0.027	1.000	1.000
FEV1/FVC	mVDS	−0.293	0.226	−0.218	−1.297	0.203	−0.751	0.165	1.000	1.000
FVC	mVDS	−0.425	0.149	−0.480	−2.852	0.007 *	−0.726	−0.123	1.000	1.000

CI: Confidence interval. * Significant difference was noted between the two groups (*p* < 0.05). PFT, pulmonary function test; FVC, forced vital capacity; FEV1/FVC, forced expiratory volume in one second/forced vital capacity.

## Data Availability

The data presented in this study are available on request from the corresponding author.
